# Arthroscopic reduction and internal fixation (ARIF) for talar body fractures: systematic review

**DOI:** 10.1051/sicotj/2023017

**Published:** 2023-06-23

**Authors:** Nicolas Cellier, Camille Sleth, François Bauzou, Pascal Kouyoumdjian, Remy Coulomb

**Affiliations:** 1 Orthopedic and Trauma surgery Department, Hospital and University Center of Caremeau Nîmes Rue du Professeur Robert Debré 30029 Nîmes France; 2 Clinique du Ter 5 Allée de la Clinique du Ter 56270 Ploemeur France; 3 Université Montpellier 1 2 Rue de l’École de Médecine 34090 Montpellier France; 4 Laboratoire de Mécanique et Génie Civile (LMGC), CNRS-UM1 860 Rue de St – Priest 34090 Montpellier France

**Keywords:** Talar body fracture, Arthroscopy, Percutaneous fixation, Fluoroscopy

## Abstract

*Purpose*: This study aimed to systematically assess the available literature on the technique and results of arthroscopic reduction – internal fixation for displaced fractures of the talar body. *Methods*: A systematic review was made of the available literature on MEDLINE, EMBASE, and the Cochrane Library database, including studies from January 1985 to July 2021. The literature search, data extraction, and quality assessment were conducted by two independent reviewers. Surgical technique, perioperative management, clinical outcome scores, radiographic outcomes, and complication rates were evaluated. *Results*: Out of 37 articles reviewed, 12 studies met the inclusion criteria. The studies included reported on the results of 22 patients. No complications were observed in any of the patients treated. *Conclusions*: The included studies had too many weaknesses to allow the pooling of data or meta-analysis. However, percutaneous arthroscopic talar internal fixation appears to be a good option for uncomplicated displaced intra-articular talar fractures. Appropriately powered randomized controlled trials with long-term follow-ups are required to confirm the effectiveness of this technique. Level of Evidence: Level IV, a systematic review of Level IV studies.

## Introduction

The indications for arthroscopic surgery have been increasing over the years. Arthroscopically-assisted techniques have been widely used in traumatic cases (hip, knee, and wrist) as well as foot and ankle surgery [1]. Arthroscopically-Assisted Reduction and Internal Fixation (ARIF) in acute trauma has gained popularity in foot and ankle surgery [2].

In the management of talar body fractures, the main purpose is to restore joint anatomy. Although open reduction and internal fixation (ORIF) is a common gold standard procedure, arthroscopic management has also been proposed [3]. The potential benefits of arthroscopic techniques, including less extensive exposure with minimal soft tissue damage, preservation of blood supply, better visualization of fracture fragments, and accurate joint reduction control are broadly described [1–5]. Although arthroscopy is increasingly used in the context of trauma, the effectiveness of ARIF compared to ORIF for the management of fractures of the talar body remains yet to be determined [5].

Although a few case reports and small series have been published, no systematic review has ever been made. This systematic review of the literature aims to assess the surgical technique, results and safety of ARIF for talar fractures.

## Materials and methods

### Search strategy

A systematic review of the literature in PubMed (MEDLINE), EMBASE, and the Cochrane database was made until November 2022. The search terms used were: “talar”, “fracture”, and “arthroscopy”. After the initial Medical Subject Headings keyword search, additional manual searches were conducted using the bibliographies of all selected full-text articles. Forty-one potential titles and abstracts were identified from the electronic database.

### Study selection

The time frame for the literature search was set from January 1985 to November 2021. This time frame was chosen regarding the beginning of practice and research in arthroscopic techniques for foot surgery. We decided not to include any studies published before 1985 as, from that year onwards publications on ankle arthroscopy began to increase significantly. All studies included met the following criteria: investigating humans treated by ARIF, papers published in English, including at least one patient for a minimum follow-up of 6 months and reporting outcome measures relating to pain or function outcome, radiographic evaluation and complication rates. Percutaneous fixation was defined as the use of stab incisions and direct insertion of screws via the skin surface. Exclusion criteria included any papers that did not meet the inclusion criteria, as well as those that included patients with an extensive approach. Study selection consisted of first reviewing the titles and abstracts of studies meeting the inclusion criteria, then scanning the selected studies with their full text.

### Data extraction

Two reviewers extracted data independently using a predefined data extraction form (visual human reading of papers). Data included demographic information, the methodology, Hawkins and Sneppen classifications [6, 7], details on surgeries protocol and reported outcomes. Reported clinical outcomes using the Ankle-Hindfoot Scale developed by the American Orthopaedic Foot and Ankle Society (AOFAS) [8] were assessed if reported at the last follow-up. Complications included superficial and/or deep infections, implant removal, misplaced screw, conversion to an open technique, additional injury revealed by arthroscopy, nerve injury, pseudarthrosis, and evolution to subtalar arthrosis were assessed if reported. Reported preoperative computed tomography (CT) evaluations made it possible to analyze and classify fractures.

The radiological evaluation of talar body fracture was based on the Sneppen classification [7]: type I were compression fractures, type II were coronal, sagittal, or horizontal plane shearing fractures, type III was posterior tubercula fractures; type IV were lateral tubercule fractures, and type V was crush fractures.

Neck fracture analysis was based on the Hawkins classification [6]: type I fractured without displacement, type II were fractures with subtalar dislocation, type III with subtalar and tibiotalar dislocation, and type IV associated with a talonavicular dislocation. Assessment of bone fracture healing if reported was made at last the follow-up.

### Evaluation of study quality

The methodological quality of each study was assessed via the MINORS score, a methodological index for evaluating non-randomized studies [9]. The exact criteria assessed are reported in Table 1. Studies with a MINORS score over or equal to 75% were considered as being at a low risk of bias. Studies with a MINORS score lower than 75% were considered as being at a high risk of bias.

**Table 1:** Minors scores for each study to assess methodological quality.

Authors	Score/16	Risk of bias	1	2	3	4	5	6	7	8
Saltzman et al. [11]	10	High	2	2	0	2	1	1	2	0
Monllau et al. [12]	11	High	2	2	0	2	1	2	2	0
Subairy et al. [13]	8	High	2	2	0	2	0	0	2	0
Ogut et al. [15]	11	High	2	2	1	2	0	2	2	0
Dodd et al. [14]	11	High	2	2	0	2	1	2	2	0
Sitte et al. [16]	10	High	2	2	1	2	0	1	2	0
Wajsfisz et al. [17]	12	Low	2	2	2	2	0	2	2	0
Kadakia et al. [18]	12	Low	2	2	2	2	0	2	2	0
Hama et al. [19]	12	Low	2	2	2	2	0	2	2	0
Wagener et al. [20]	12	Low	2	2	2	2	0	2	2	0
Oliveira et al. [21]	8	High	2	2	0	2	0	0	2	0
Bardas et al. [22]	12	Low	2	2	2	2	0	2	2	0

Numbers signification: 1. A clearly stated aim; 2. Inclusion of consecutive patients; 3. Prospective collection of data; 4. Endpoints appropriate to the aim of the study; 5. Unbiased evaluation of the study endpoint; 6. Follow-up period appropriate to the aim of the study; 7. Loss to follow-up less than 5%; 8. Prospective calculation of the study size. *The final score comprises the results of 8 items or 12 items in the case of comparative studies.*

### Data analysis

This systematic review was reported by the Preferred Reporting Items for Systematic Reviews and Meta-Analyses (PRISMA) statement [10]. Data were extracted from the papers by systematic analysis of each article and summarization (Microsoft Excel version 2010, Microsoft, Redmond, WA, USA).

## Results

**Figure 1:**
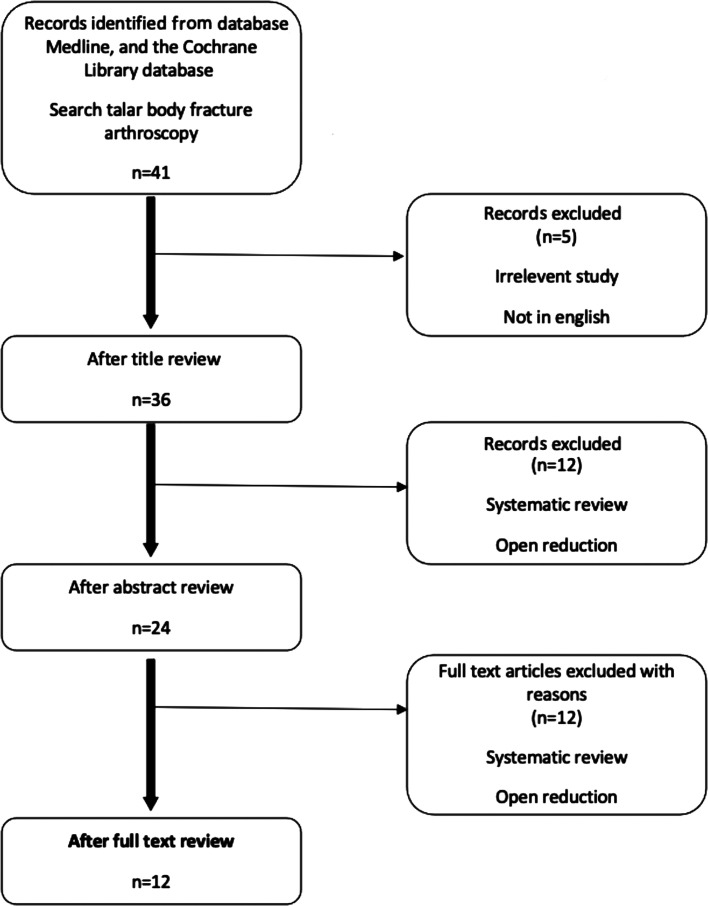
Search strategy. PRISMA flow diagram. PRISMA, preferred reporting items for systematic reviews and meta-analyses.

The results of the search strategy and study selection criteria are shown in Figure 1. A total of 12 studies were included in this systematic review [11–22].

### Population characteristics

The twelve studies collected a total of 22 patients. Fifteen of them were men (68%). The average age ranged from 14 to 61 years. The average follow-up period varied from 12 to 36 months. Ten studies included Sneppen Type II fractures [11, 13, 14, 16–22] and two studies included Sneppen type III [12, 15]. Eleven studies included Hawkin’s type II fractures [11–13, 15–22] and only one Hawkin’s type III [17]. Demographic details are shown in Table 2.

**Table 2:** Main characteristics of included studies with arthroscopic assisted reduction internal on displaced talar body fractures.

Authors	Study design/Level of evidence	Nb patients	Mean age (years)	Follow-up (month)	Hawkins/Sneppen type	Clinics scores at last follow-up	Complications
Saltzman et al. [11]	CR, V	1	25	6	II/II	NR	0
Monllau et al. [12]	CR, V	1	29	12	II/III	NR	0
Subairy et al. [13]	CR, V	1	15	NR	II/II	NR	0
Ogut et al. [15]	CR, V	1	24	12	II/III	NR	0
Dodd et al. [14]	CR, V	1	19	12	NR/II	NR	0
Sitte et al. [16]	IV	2	29	6	II/II	AOFAS (75)	0
Wajsfisz et al. [17]	CR, V	1	17	29	III/II	AOFAS (97)	0
Kadakia et al. [18]	CR, V	1	29	12	II/II	FADI (85.6)	0
Hama et al. [19]	CR, V	1	14	12	II/II	JOA (95)	0
Wagener et al. [20]	IV	7	39	30	II/II	VAS (0.1)	0
Oliveira et al. [21]	CR, V	1	22	6	II/II	AOFAS (85)	0
Bardas et al. [22]	IV	4	38	18	II/II	AOFAS (92.8)	0

Abbreviations: AOFAS, American Orthopaedic Foot and Ankle Society; CR, case report; FADI, Foot and Ankle Disability Index; JOA, Japanese Orthopaedic Association; NR, not recorded; VAS, Visual analogue scale.

### Study quality

Using the MINORS scale, the assessment of the methodological quality resulted in a mean score of 10.8/16 (maximum score of 12/16) for non-comparative studies (Table 1). Three studies were level IV of evidence [16, 20, 22] and nine of level V [11–15, 17–19, 21].

### Surgical techniques (Table 3)

**Figure 2:**
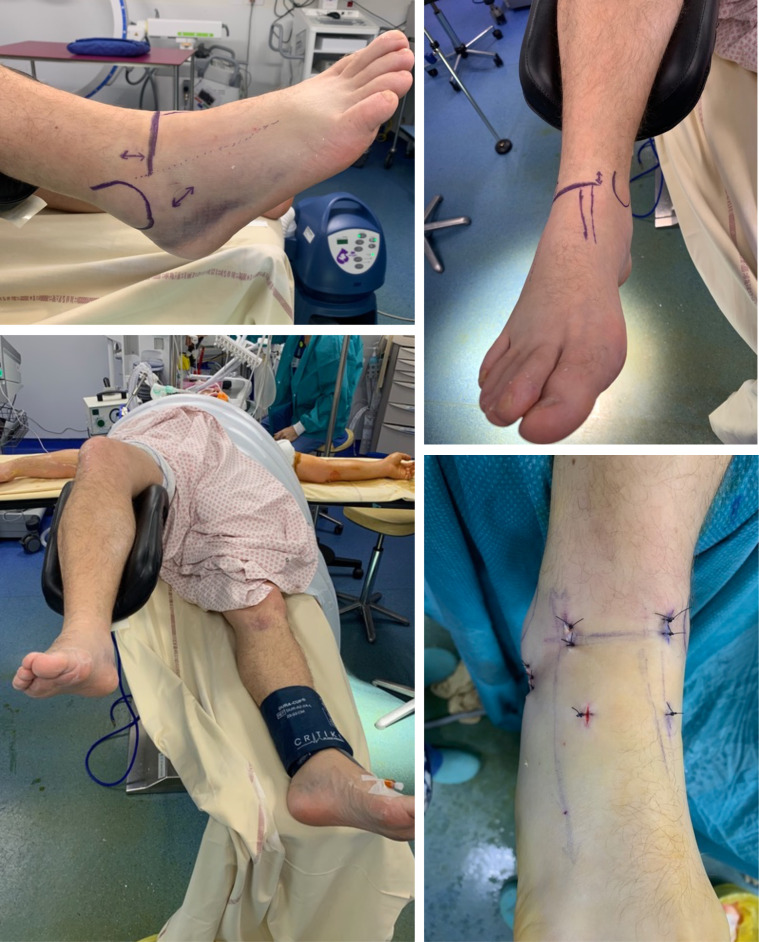
Installation, approaches, and incision for anterior-posterior cannulated screw osteosynthesis.

**Figure 3:**
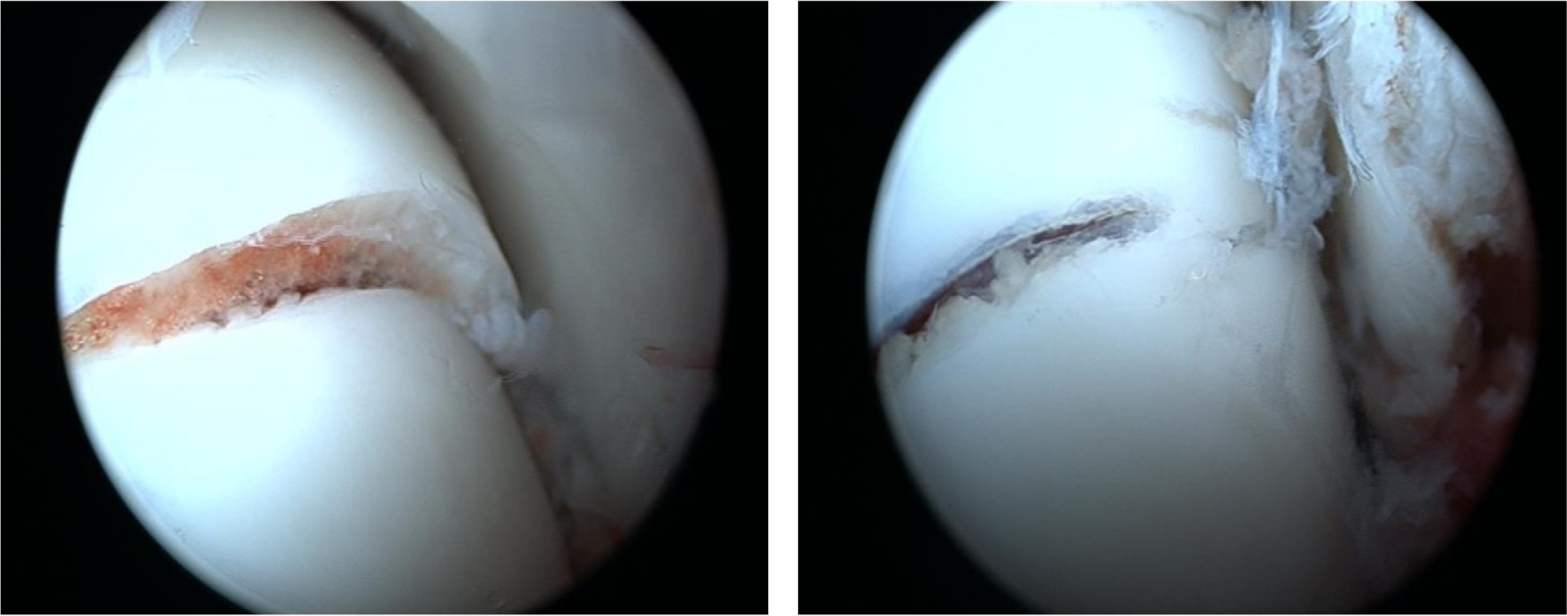
Preoperative view before and after reduction, without compression.

**Figure 4:**
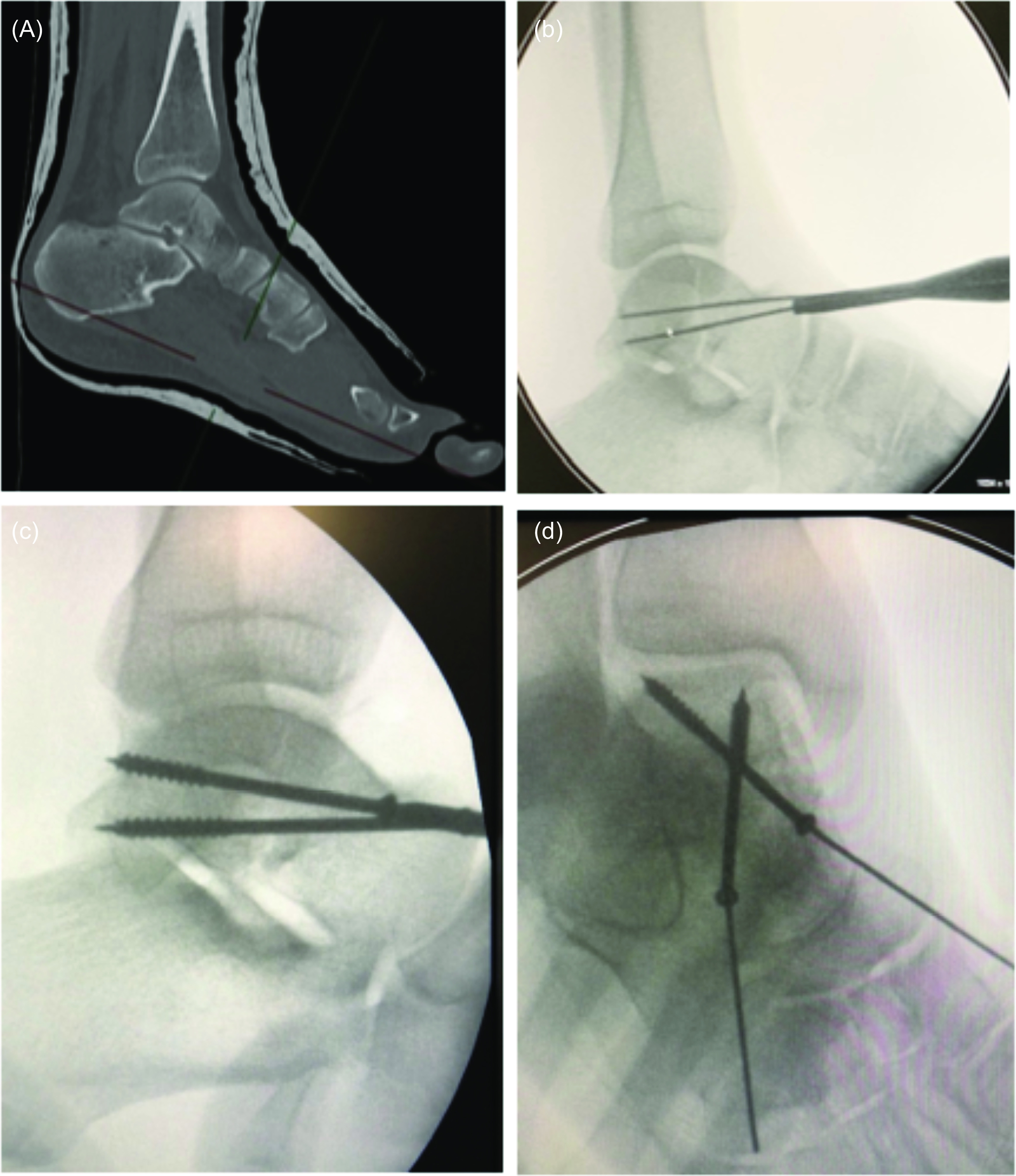
Preoperative scan assessment (A), intraoperative fluoroscopy (B, C, D).

Surgery was usually performed under general anaesthesia. Patients were always in a supine position. All studies exclusively described a percutaneous approach. The arthroscopy was performed with a classical anteromedial and anterolateral portal (Figure 2). An accessory portal was used in some cases (most of the time directly over the sinus tarsi). A small diameter arthroscope (2.7–4 mm/0–30°) and a small shaver were used because of the narrow space in the ankle and subtalar joint. No distraction devices were used for arthroscopy. If there was a displaced fracture or interposed fragment in the subtalar joint on the preoperative radiographs, arthroscopy was performed through a sinus tarsi portal for debridement and/or reduction. In all patients, a Kirschner wire (K-wire) was placed, under fluoroscopic control, distal to the fracture in the medial aspect of the talus head, to facilitate reduction, using the K-wire as a joystick. The reduction was verified by both fluoroscopy and arthroscopy (Figure 3) and additional K-wires were used on more complex fractures. Percutaneous osteosynthesis was most frequently performed with two cannulated cortical screws (Figure 4).

**Table 3:** Surgical techniques on studies with arthroscopic assisted reduction internal on displaced talar body fractures.

Authors	Time from injury to surgery (day)	Optic (mm)	Portals	Operative times (min)	Immobilization (week)	Duration of non-weight bearing (week)	Mean postoperative hospital stay (day)
Saltzman et al. [11]	1	2.7	AM/AL	NR	2	12	10
Monllau et al. [12]	NR	NR	AM/AL	NR	2	12	NR
Subairy et al. [13]	1	NR	AM/AL	NR	NR	NR	NR
Ogut et al. [15]	NR	4	PL/PM	NR	7	10	NR
Dodd et al. [14]	NR	NR	AM/AL	NR	8	8	NR
Sitte et al. [16]	45–270	2.7/4	PL/PM/C/AL	98	0	8	NR
Wajsfisz et al. [17]	NR	NR	AM/AL/ST	NR	6	6	NR
Kadakia et al. [18]	NR	4	PL/PM	NR	6	6	NR
Hama et al. [19]	NR	NR	NR	NR	12	12	NR
Wagener et al. [20]	1–47	4	C/ST	59	8	6	2-9
Oliveira et al. [21]	NR	4	AM/AL	NR	8	8	NR
Bardas et al. [22]	1	NR	AM/AL/ST	NR	12	12	NR

Abbreviations: AARIF, Arthroscopic assisted reduction internal; AL, antero-lateral; AM, antero-medial C, central; NR, not recorded; PL, postero-lateral; PM, postero-medial; ST, subtalar.

### Hospital stay and post-operative instructions

The mean postoperative hospital stay was reported in three studies and ranged from 2 to 10 days. Early passive mobilization was performed on the first postoperative day. Some authors allowed partial weight-bearing during the first 6 weeks associated with an extensive physiotherapy program including active range of motion (ROM) exercises and passive mobilization. Some authors reported the use of elastic dressing [11, 12, 16] or a simple cast [15, 18, 20–22]. Full weight-bearing was generally allowed 6–8 weeks after surgery.

### Clinical scores

Only four studies [16, 17, 21, 22] reported AOFAS scores [8]. The average ranged from 75 to 97 at the last follow-up. One study [18], reported the foot and ankle disability index score (85.6/100) [23], one [19] the Japanese orthopaedic association score (96/100) [24], and one a visual analogue scale (mean value 0.1/10) [20] at last follow-up.

### Radiological evaluation

Most of the studies performed a postoperative X-ray control and two studies reported the use of magnetic resonance imaging (MRI) to assess the talus vascularization 3 months after surgery. Two studies reported Hawkins’ signs seen in anterior-posterior (AP) view that described the subchondral lucency of the talar dome secondary to subchondral atrophy 6–8 weeks after a talar neck fracture. This sign indicates sufficient blood supply to the talus, making it less probable that it will develop into avascular necrosis [6, 25].

### Complications

No wound dehiscence or superficial infection was described. No postoperative soft tissue complications have been linked to this technique. No studies mentioned any nerve damage and no surgical revisions were reported. The bone healing rate noticed in this review was 100% with no pseudarthrosis but only one study [12] described a second-look arthroscopy at 3 months postoperatively and showed complete consolidation. However, no systematic CT scan or MRI monitoring to assess fusion was performed. No avascular necrosis of the talus was reported and no subtalar fusion was required at the end of the follow-up period in the studies [4, 11–22]. Regarding osteoarthritis, no data was collected. That can be explained by a too-brief follow-up of the studies in this review. In fact, the development of post-traumatic arthritis of the ankle or subtalar occurs after more than 30 months. The longest follow-up was reported by Wagener et al. [20] at 30 months.

## Discussion

Arthroscopy has been used for many indications in traumatic surgery, to diagnose and monitor the reduction of many types of joint fractures [1]. The use of arthroscopic techniques for ankle and subtalar displaced fracture treatment avoids the large surgical approaches, wound dehiscence and infection often encountered with open surgical techniques. It also allows assessments of the accuracy of reduction. The decrease in overall morbidity related largely to smaller and sparing approaches has led to an expanded role for arthroscopy in the treatment of foot and ankle injuries with the use of dedicated arthroscopic-assisted open reduction internal fixation [1].

Most talar fractures are articular and the prognosis is related to anatomical reduction. Changes in physiological foot geometry are poorly tolerated [26]. This type of failure is usually related to poor preoperative radiographic evaluation of the fracture and its poor reduction and/or stabilization. The surgical treatment of talar neck fractures is associated with a high complication rate, including malunion, nonunion, and avascular necrosis [27, 28]. The good clinical results of this review could be related to better fracture reduction and joint congruency thanks to the use of dual-modality imaging (fluoroscopy and arthroscopy). No post-traumatic talar avascular necrosis was found in this review. Arthroscopic soft tissue preservation may be a protective factor [29].

There are several factors to consider in determining whether arthroscopic management is appropriate or not. Characterization of the fracture is one of the main points to consider [18]. Thus, this review highlights the viability of the arthroscopic-assisted reduction with a percutaneous fixation for the treatment of Hawkins type II or III talar neck fractures. Most of the reported patients presented a Hawkins type II fracture. Wagener et al. [20] conclude that the most appropriate lesion for treatment by percutaneous fixation with dual-modality imaging is Hawkins type II fractures with limited comminution. No information and cases were reported for the other type of fracture in order to evaluate the viability of the arthroscopic assistance in the other specific cases. Nevertheless, the use of arthroscopy for talar fracture is case-specific and surgeon-dependent as discussed in Bonasia et al.’s study [4].

A further benefit of arthroscopic treatment is the detection of additional injuries that may have been missed with percutaneous or open techniques. These concomitant injuries can be addressed in the same operation. Sadamasu et al. [30] published an incidence of 30% of peroneal tendon dislocations in their series of 30 talar fractures detected on a pre-operative CT scan. It is not possible to diagnose and treat these lesions with only an ankle arthroscopy procedure.

The ARIF technique can be used on patients with surgical risk factors (smokers, diabetics, etc), probably for open fractures and for patients with soft tissue damage like general swelling or diffuse hematoma. This type of surgical technique must be performed early on after the injury to allow percutaneous mobilization of the fracture.

Some authors [4, 19] have emphasized the difficulty of managing tibiotalar and subtalar arthroscopies without iatrogenic chondral lesions. For those authors, the use of a small diameter arthroscope (2.4 mm) was necessary, without a distraction system. In the learning curve, temporary use of an external fixator has also been reported. When percutaneous methods fail or if there are substantial bone impactions, additional portals or reverting to a small incision (sinus tarsi approach) may be considered [16–18, 20, 21]. None of the studies reported reverting to open surgery.

We attempted to conduct a systematic review looking at the role of arthroscopic-assisted reduction in the surgical fixation of fractures of the neck and body of the talus. This is a reasonable clinical question, because fractures of the talus are associated with high rates of long-term morbidity, even with expert fixation. Previous literature suggests that poor clinical results are associated with malreduction, and to a lesser extent avascular necrosis.

This systematic review has some limitations, including insufficient literature to support definitive conclusions. All of the studies included are case reports or case series with a low level of evidence and a small number of patients. There is too much heterogeneity in fracture types, post-operative treatment, outcome measures, duration of follow-up with an average of 14.1 months and radiological assessment. Therefore, it seems difficult to conclude mid-term complications or the reliability of consolidation or avascular necrosis rate. The only real conclusion is there is a need for good-quality outcomes research, but with such a rare injury this is unlikely to be possible at a single centre. Suggesting a randomized controlled trial is useless because it is impossible to perform under these conditions. More likely it would need to be a registry-based project, and there needs to be a comparison group of patients who underwent “standard” open reduction and internal fixation.

## Conclusion

The use of arthroscopic assistance seems to be a fair option to consider for the treatment of displaced talar fractures with a low complication rate. Appropriately powered trials and long-term follow-up are required to assess the effectiveness of ARIF techniques compared to open reduction and internal fixation.

## References

[1] Hamilton GA, Doyle MD, Castellucci-Garza FM (2018) Arthroscopic-assisted open reduction internal fixation. Clin Podiatr Med Surg 35, 199–221. 29482790 10.1016/j.cpm.2017.12.004

[2] van Dijk NC, van Bergen CJA (2008) Advancements in ankle arthroscopy. J Am Acad Orthop Surg 16, 635–646. 18978286 10.5435/00124635-200811000-00004

[3] Buza JA, Leucht P (2018) Fractures of the talus: current concepts and new developments. Foot Ankle Surg 24, 282–290. 29409210 10.1016/j.fas.2017.04.008

[4] Bonasia DE, Rossi R, Saltzman CL, Amendola A (2011) The role of arthroscopy in the management of fractures about the ankle. J Am Acad Orthop Surg 19, 226–235. 21464216 10.5435/00124635-201104000-00007

[5] Gonzalez TA, Macaulay AA, Ehrlichman LK, Drummond R, Mittal V, DiGiovanni CW (2016) Arthroscopically assisted versus standard open reduction and internal fixation techniques for the acute ankle fracture. Foot Ankle Int 37, 554–562. 26660864 10.1177/1071100715620455

[6] Hawkins LG (1970) Fractures of the neck of the talus. J Bone Joint Surg Am 52, 991–1002. 5479485

[7] Sneppen O, Christensen SB, Krogsoe O, Lorentzen J (1977) Fracture of the body of the talus. Acta Orthop Scand 48, 317–324. 920125 10.3109/17453677708988775

[8] Kitaoka HB, Alexander IJ, Adelaar RS, Nunley JA, Myerson MS, Sanders M (1994) Clinical rating systems for the ankle-hindfoot, midfoot, hallux, and lesser toes. Foot Ankle Int 15, 349–353. 7951968 10.1177/107110079401500701

[9] Slim K, Nini E, Forestier D, Kwiatkowski F, Panis Y, Chipponi J (2003) Methodological index for non-randomized studies (minors): development and validation of a new instrument. ANZ J Surg 73, 712–716. 12956787 10.1046/j.1445-2197.2003.02748.x

[10] Moher D, Liberati A, Tetzlaff J, Altman DG, Group PRISMA (2010) Preferred reporting items for systematic reviews and meta-analyses: the PRISMA statement. Int J Surg 8, 336–341. 20171303 10.1016/j.ijsu.2010.02.007

[11] Saltzman CL, Marsh JL, Tearse DS (1994) Treatment of displaced talus fractures: an arthroscopically assisted approach. Foot Ankle Int 15, 630–633. 7849981 10.1177/107110079401501111

[12] Monllau JC, Pelfort X, Hinarejos P, Ballester J (2001) Combined fracture of the talus: arthroscopic treatment. Arthroscopy 17, 418–421. 11288018 10.1053/jars.2001.21847

[13] Subairy A, Subramanian K, Geary NPJ (2004) Arthroscopically assisted internal fixation of a talus body fracture. Injury 35, 86–89. 14728962 10.1016/s0020-1383(02)00374-1

[14] Dodd A, Simon D, Wilkinson R (2009) Arthroscopically assisted transfibular talar dome fixation with a headless screw. Arthroscopy 25, 806–809. 19560647 10.1016/j.arthro.2009.01.002

[15] Ogut T, Seyahi A, Aydingoz O, Bilsel N (2009) A two-portal posterior endoscopic approach in the treatment of a complex talus fracture: a case report. J Am Podiatr Med Assoc 99, 443–446. 19767553 10.7547/0990443

[16] Sitte W, Lampert C, Baumann P (2012) Osteosynthesis of talar body shear fractures assisted by hindfoot and subtalar arthroscopy: technique tip. Foot Ankle Int 33, 74–78. 22381240 10.3113/FAI.2012.0074

[17] Wajsfisz A, Makridis KG, Guillou R, Pujol N, Boisrenoult P, Beaufils P (2012) Arthroscopic treatment of a talar neck fracture: a case report. Knee Surg Sports Traumatol Arthrosc 20, 1850–1853. 22048748 10.1007/s00167-011-1742-3

[18] Kadakia R, Konopka J, Rodik T, Ahmed S, Labib SA (2017) Arthroscopic reduction and internal fixation (ARIF) of a comminuted posterior talar body fracture: surgical technique and case report. Foot Ankle Spec 10, 465–469. 28068792 10.1177/1938640016685148

[19] Hama S, Onishi R, Yasuda M, Minato K, Miyashita M (2018) Adolescent talus body fracture with high displacement: a case report. Medicine 97(35), e12043. 30170415 10.1097/MD.0000000000012043 PMC6393032

[20] Wagener J, Schweizer C, Zwicky L, Horn Lang T, Hintermann B (2018) Arthroscopically assisted fixation of Hawkins type II talar neck fractures: a case series. Bone Joint J 100, 461–467. 29629582 10.1302/0301-620X.100B4.BJJ-2017-0772.R3

[21] Oliveira MA, Sousa H, Ventura M, Oliveira JR, Sá D, Lemos C (2020) Arthroscopically assisted reduction and internal fixation of talar neck fracture: a case report. J Orthop Case Rep 9(6), 90–93. 32548038 10.13107/jocr.2019.v09.i06.1604 PMC7276598

[22] Bardas CA, Benea HRC, Apostu D, Oltean-Dan D, Tomoaia G, Bauer T (2021) Clinical outcomes after arthroscopically assisted talus fracture fixation. Int Orthop 45(4), 1025–1031. 33078205 10.1007/s00264-020-04859-5

[23] Martin R, Burdett RB, Irrgang J (1999) Development of the foot and ankle disability index (FADI). J Orthop Sports Phys Ther 29, 32–33.

[24] Niki H, Aoki H, Inokuchi S, Ozeki S, Kinoshita M, Kura H, Tanaka Y, Noguchi M, Nomura S, Hatori M, Tatsunami S (2005) Development and reliability of a standard rating system for outcome measurement of foot and ankle disorders I: development of standard rating system. J Orthop Sci 10(5), 457–465. 16193356 10.1007/s00776-005-0936-2 PMC2797841

[25] Tezval M, Dumont C, Stürmer KM (2007) Prognostic reliability of the Hawkins sign in fractures of the talus. J Orthop Trauma 21, 538–543. 17805020 10.1097/BOT.0b013e318148c665

[26] Suter T, Barg A, Knupp M, Henninger H, Hintermann B (2013) Surgical technique: talar neck osteotomy to lengthen the medial column after a malunited talar neck fracture. Clin Orthop Relat Res 471, 1356–1364. 23073707 10.1007/s11999-012-2649-0 PMC3586008

[27] Canale ST, Kelly FB (1978) Fractures of the neck of the talus. Long-term evaluation of seventy-one cases. J Bone Joint Surg Am 60, 143–156. 417084

[28] Rammelt S, Zwipp H (2009) Talar neck and body fractures. Injury 40, 120–135. 18439608 10.1016/j.injury.2008.01.021

[29] Prasarn ML, Miller AN, Dyke JP, Helfet DL, Lorich DG (2010) Arterial anatomy of the talus: a cadaver and gadolinium-enhanced MRI study. Foot Ankle Int 31, 987–993. 21189192 10.3113/FAI.2010.0987

[30] Sadamasu A, Yamaguchi S, Nakagawa R, Kimura S, Endo J, Akagi R (2017) The recognition and incidence of peroneal tendon dislocation associated with a fracture of the talus. Bone Joint J 99, 489–493. 28385938 10.1302/0301-620X.99B4.BJJ-2016-0641.R1

